# Defensive Aggregation (Huddling) in *Rattus Norvegicus* toward Predator Odor: Individual Differences, Social Buffering Effects and Neural Correlates

**DOI:** 10.1371/journal.pone.0068483

**Published:** 2013-07-29

**Authors:** Michael T. Bowen, Richard C. Kevin, Matthew May, Lauren G. Staples, Glenn E. Hunt, Iain S. McGregor

**Affiliations:** 1 School of Psychology, University of Sydney, Sydney, New South Wales, Australia; 2 Department of Psychology, Macquarie University, Sydney, New South Wales, Australia; 3 Discipline of Psychiatry, Concord Clinical School, University of Sydney, Sydney, New South Wales, Australia; Sapienza University of Rome, Italy

## Abstract

Aggregation is a defensive strategy employed by many prey species in response to predatory threat. Our group has characterized defensive aggregation (huddling) in *Rattus norvegicus* in response to a ball of cat fur. In this situation some rats huddle less, and approach the threatening cue more than others (active vs. passive responders). The present study explored whether active responding is a stable phenotype associated with behaviors outside direct predatory encounters. The neural substrates of active and passive responding under predatory threat were explored using c-Fos immunohistochemistry. Finally, we examined whether the presence of conspecifics during predatory threat biases behavior towards active responding. Active and passive responding styles were found to be stable in individual rats across consecutive group exposures to cat fur, and were predicted by anxiety-like behavior in an open-field emergence test. Active responders displayed less conditioned fear in an environment associated with predatory threat, and had higher post-exposure intake of a weak sucrose solution (a test of “anhedonia”). Active responding was associated with: greater cat fur-induced activation of the accessory olfactory bulb, reflecting greater olfactory stimulation in rats actively approaching the fur; lowered activation of somatosensory cortex, reflecting reduced huddling with conspecifics; and reduced activation in the lateral septum. Social exposure to cat fur promoted active responding relative to individual exposure, and lowered c-Fos expression in the dorsomedial periaqueductal grey, medial caudate putamen and lateral habenula. We conclude that individual differences in anti-predator behavior appear stable traits with active responders having a more resilient phenotype. Social exposure to predatory threat has an acute buffering effect, subtly changing the neural and behavioral response towards threat and encouraging active responding. An association between active responding and lower c-Fos expression in the lateral septum is consistent with previous studies that highlight this region as an important neurobiological substrate of defensive aggregation.

## Introduction

Defensive aggregation is a ubiquitous response in prey species involving the tight grouping (e.g. huddling, flocking or shoaling) of animals in response to predatory threat. Defensive aggregation is observed across a wide array of mammalian and non-mammalian species [1], for example: Emperor Penguins [2]; the marine insect *Halobates robustus* [3]; and Serengeti ungulates such as wildebeest, zebra, and Thomson’s gazelle [4]. Investigating the behavioral dynamics and neurobiological underpinnings of defensive aggregation is of increasing interest to researchers in both the laboratory and the field (e.g. [5]–[10]).

Laboratory models of defensive aggregation allow systematic study of the neural substrates, pharmacology and genetic determinants of this behavioral phenomenon. Our own research has focused on groups of laboratory rats confronted with predatory threat in the form of a ball of cat fur. Cat fur and skin odors cause characteristic behavioral and neural changes in individual rats that are indicative of a profound anxiety-like state (for a review see [11]). The response of groups of laboratory rats to predatory threat was first described by Blanchard & Blanchard [12] using a laboratory “visible burrow system”. These authors reported hasty retreat and a subsequent increase in non-sexual, non-aggressive social contacts in the burrow system when rats encountered a live cat at the burrow surface. In more recent work [13], [14], our group has described high levels of huddling when groups of four cage mates are exposed to cat fur in an open arena. This does not occur, however, when only two rats are present [13]. Such results are predicted by the *dilution effect,* which sees the survival benefit afforded by aggregation as increasing with the size of the group, through a decrease in the probability that any individual animal will be attacked [3], [15].

Cat fur/skin odor appear to function as *kairomones*: defined as chemosensory signals that are emitted by one species and intercepted by another to the advantage of the recipient [16]. In other words, the sensitivity of rodents to cat fur confers a survival advantage, and they are most likely intercepting signals that cats use in their own social communication. Studies using c-Fos immunohistochemistry show that cat odor activates pheromone processing circuitry in the rat brain localised within the accessory olfactory bulb (AOB) and its projection areas in the medial amygdala, bed nucleus of the stria terminalis, and ventromedial hypothalamus [17]–[19]. A medial hypothalamic circuit, with the dorsal premammillary nucleus (PMD) at its center, integrates this sensory input and organizes behavioral output via projections to the periaqueductal gray (PAG) and cuneiform nucleus. This system organises behavioral responses that may include escape attempts, immobility and inhibition of feeding, foraging and reproduction – all of which are characteristic responses to predatory threat [11], [17], [18], [20]–[22].

Our informal observations of huddling in outbred laboratory rats exposed to predatory threat [13], [14] suggests divergent response styles in the individuals within each group of four rats exposed to cat fur. Some rats (“active responders”) show relatively high levels of investigation of the cat fur, and relatively low levels of immobility and huddling with conspecifics. In contrast, many rats are “passive responders” and barely investigate the threatening stimulus, showing high levels of immobility and huddling. This is in accord with previous suggestions of active and passive responder styles in outbred rats exposed to other forms of stress [23]. Active responders proactively confront threats, have a more aggressive phenotype, and show less immobility and hypothalamic-pituitary-adrenal (HPA) axis activity and reactivity compared to their passive counterparts. Conversely, passive responders avoid threats, have a less aggressive phenotype, and respond actively only when it is absolutely necessary [24], [25].

Active and passive coping styles may have emerged as adaptations to the changing environments that animals experience in the wild [26]. For example, Dingemanse and colleagues [26] studied a population of *Parus major* and found that in years when food was plentiful, active responding males had greater rates of survival. Conversely, in years when food was scarce, more passive responding males had greater rates of survival. The enhanced ability of active responding males to secure and hold onto territory in resource-rich years improves their chances of survival. Conversely, their more overtly aggressive and territorial phenotype in resource-scarce years may result in more net costs than benefits. Interestingly, the relationship between responder type, availability of resources, and rate of survival was the opposite for females, further driving diversity in offspring responder types [26].

In the present study, our initial aim was to examine whether active or passive responding to predatory threat was a stable behavioral trait in laboratory rats. As such, we individually marked rats and then scored their approach and huddling response to cat fur over consecutive exposures in groups. We also examined whether the passive versus active responder style was associated with behavioral differences in situations other than cat fur exposure, including the open field emergence test [27] and an environment previously associated with predatory threat [28]. We also examined whether active and passive responders differed in their consumption of a weak 1% sucrose solution, a marker of anhedonia, or depressive-like behavior, in rats [29]. Our highly developed knowledge of the patterns of neural activation elicited in rats individually exposed to cat odor [17]–[19], [30] also allowed an opportunity to examine how active and passive responding may manifest itself at a neural level. In this regard we used c-Fos immunohistochemistry to determine neural activation to cat fur in rats exhibiting an active or passive coping style.

We also hypothesised that exposure to threat in a group may encourage a more active style of coping, by reducing the acute anxiety arising from the experience. The term “social buffering” describes how social interaction with conspecifics during or after exposure to a stressor may reduce the impact of that stressful situation [31]. A small number of studies with rodents suggest that the behavioral, physiological and neural responses to stressors may be reduced by the presence of conspecifics either during or after a stressor such as shock [31], [32]. To examine possible social buffering effects, we compared behavior and brain activation in rats exposed to cat fur when alone, with those exposed in a group of four conspecifics. We predicted that group exposure might provide some buffering effects, resulting in a less pronounced neural and behavioral response to cat odor in the socially exposed rats.

In summary, the primary aims of the present study were twofold. The first aim was to formally examine the proposition that within a group of rats exposed to cat odor there are active and passive responders and that these characteristics are relatively stable over repeated exposure to threat, and associated with differential neural activation. The second aim was to characterize how group exposure to predatory threat differs from individual exposure, and whether social buffering might promote a more active style of responding.

## Methods

All experimental procedures were conducted in accordance with the Australian Code of Practice for the Care and Use of Animals for Scientific Purposes (7^th^ Edition, 2004) and were approved by University of Sydney Animal Ethics Committee (approval number L29/7-2010/3/5360).

### 2.1. Experiment 1

#### 2.1.1. Aims

**Figure 1:**
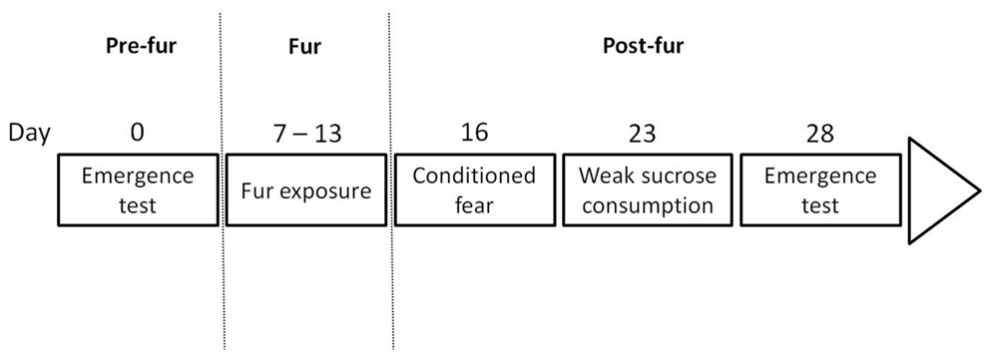
Timeline for experiment 1. An emergence test was initially conducted prior to cat fur exposure. Rats were exposed to cat fur in groups of four once per day for 7 days. Three days after the final fur exposure rats were returned to the test arena individually with a hide box present but no fur present. This allowed testing for conditioned fear to context. Ten days after the final fur exposure rats were tested in a 10 min session for their consumption of a 1% sucrose solution. Finally, 15 days after the final fur exposure the emergence test was once again conducted to test for chronic stress-induced changes in anxiety-like behavior.

Experiment 1 examined the stability of active and passive response styles in groups of four rats repeatedly exposed to cat odor in daily sessions. A secondary aim was to see if responder style could be predicted from behavior in other situations, in which immediate predatory threat was not present. An overview of the tests conducted and their timeline is provided in Figure 1.

#### 2.1.2. Subjects

The subjects were 48 male outbred Albino Wistar rats (Animal Resources Centre, Perth, Western Australia) weighing between 291 and 386 g and aged 8 weeks at the start of testing. They were housed in groups of 4 in a temperature controlled colony room (21±2°C) on a reverse light-dark cycle (lights on 21∶00). Food and water were available *ad libitum* in the home cage and all behavioral testing took place during the dark cycle. Following their arrival at the laboratory, rats were thoroughly handled daily for 3 days before the start of testing. Subjects were randomly assigned to either a no cat odor control condition (n = 16, consisting of 4 quads) or a cat odor condition (n = 32, consisting of 8 quads).

#### 2.1.3. Fur exposure

*Cat odor stimulus:* The predator stimuli used were 2 g balls of cat fur acquired from male cat carcasses kindly provided by *Australian Feral Pest Management*. Feral cats are routinely shot in Australia due to the threat they pose to native Australian wildlife. Deceased cats were stored at −20°C. To prepare each predator stimulus, a 2 g ball of fur was shaved from the back and neck of the cat. The fur sample was stored in an airtight jar at − 20°C when not in use. Prior to use, the cat fur was heated in a scientific oven (Binder; Crown Scientific, Australia) at 40°C for 30 min. In between testing sessions the ball was placed in the oven for 5 min at 40°C to ensure the temperature was consistent across sessions. The heating process makes the temperature of the fur close to the body temperature of a live predator.

*Apparatus:* Testing was conducted in two identical 120 cm×120 cm×90 cm (l x w x h) wooden framed arenas, located adjacent to each other. The removable arena base comprised of a 120 cm x 120 cm wire mesh sheet (0.1 cm thick wire rods, 1 cm apart) secured to a 7 cm high wooden frame. The interior walls and arena base were painted matt black. The testing room was lit with infrared light and overhead infrared-sensitive cameras fixed directly above each arena sent their outputs to two computers in an adjacent room that digitized and recorded the sessions.

*Procedure:* Rats were exposed to cat fur in groups consisting of 4 cage mates, hereafter referred to as a “quad”. Each rat in a quad was marked with a non-toxic Sharpie permanent marker each day before testing to allow ready visual identification of individual animals within a group during subsequent video scoring of behavior. Marking involved holding the rat securely and gently making a unique mark on its back with the permanent marker. Subjects were marked with either a: cross running vertically from the upper to lower back and horizontally from shoulder to shoulder; a horizontal line running from shoulder to shoulder; a vertical line running from the upper to lower back; or a circle marked in the middle of the back.

A 2 g ball of fur was placed into each arena against the center of one wall. Quads were then placed into one of the arenas opposite the fur for 20 min. Control rat quads were handled identically but with no fur placed in the arena. This process was repeated once a day for 7 days. Each quad was always exposed in the same arena and the arenas were thoroughly cleaned with 30% v/v ethanol in between each session.

Over time, rats tend to habituate to cat fur such that they will show less defensive behavior over repeated exposures [33]. To guard against habituation to the fur, the fur was exchanged for that of another cat on the fourth day. A reinstatement of defensive responding to cat fur occurs when fur from a different cat is presented to habituated rats [14], [34].

*Classification of responder type:* Subjects in the cat odor condition were designated as either a passive, neutral or active responder based on their number of contacts with the cat odor stimulus. A stimulus contact was defined as a rat placing its nose within 3 cm of the cat fur. Rats with 2 or less stimulus contacts in a 20 min session were classified as passive responders, animals with 3–4 stimulus contacts were classified as neutral responders, and rats with 5 or more stimulus contacts were classified as active responders. Criteria based assignment was used, as opposed to a simple median split, as it allowed greater equivalence of assignment thresholds between Experiment 1 and 2, and avoided some of the major issues presented by median split dichotomization [35], which might not provide adequate separation between active and passive responders. The overall classification as active, passive, or neutral was based on the average daily number of stimulus contacts over the seven exposure sessions.

*Data collection and analysis of exposure sessions:* Two of the dependent variables of interest were number of stimulus contacts and time spent in the half of the arena containing the cat fur. These behaviors were manually scored for each individual rat from the videos by an experimenter, using ODLog (Macropad Software). Data were analysed using mixed model ANOVA and planned contrasts were computed using the values of the dependent variables averaged across the 7 exposures to compare: (1) the cat odor exposed rats versus the control rats; and (2) the active responders versus the passive responders within the cat odor-exposed cohort.

The number of fecal boli present in the arena were counted after each session: obviously this was a group measure given that fecal boli could not be attributed to individual rats. The total number of fecal boli, averaged over the 7 sessions, were compared for control quads versus fur exposed quads using a planned contrast following a mixed model ANOVA.

Huddling behavior was scored automatically using MotMen Social Tracker 2.7 (as used in [13], [14]) and was defined as three or four rats clumping together in a single tight group with each rat touching at least one other rat. The time that control quads spent huddling on each of the 7 exposure days was compared to fur-exposed quads using a planned contrast following mixed model ANOVA.

Consistency of responder type (active or passive) and huddling behavior across the 7 repeated exposures were assessed by computing Cronbach’s alpha (Cronbach’s α) and the Intraclass Correlation (ICC) using reliability analysis with a two-way random model examining absolute agreement and average measures. Cronbach’s α is a measure of internal consistency, with a Cronbach’s α >0.9 considered an indication of excellent internal consistency [36]. The ICC assesses the level of agreement between repeated ratings, with an ICC >0.8 indicating excellent agreement [37]. Essentially, these statistics indicate the consistency with which a rat was classified as the same responder type over the 7 days, and how consistent each quads huddling time was across the 7 repeated exposures (see [38]). Consistency of responder type indicates their classification as active or passive remains stable across the repeated exposures, and consistency of huddling behavior indicates the amount of time a quad spends huddling remains consistent across the repeated exposures.

#### 2.1.4. Post exposure test 1: Conditioned fear

Three days after the final cat fur (or control) exposure, individual rats were returned to the same testing arena but with no fur present. A wooden hide box with a red Perspex lid (40×24×17 cm), identical to that used in the emergence test (see below), was placed against the center of one wall. The 5 min session started with an individual rat being placed in the hide box. The following dependent variables were automatically scored by Trackmate 5.5 video tracking software (Motion Mensura, Cook’s Hill, NSW): (i) time in hide box, (ii) latency to emerge, and (iii) distance travelled. After each test the arena was cleaned with 30% v/v ethanol. Data were analysed using one-way ANOVA with four levels of the independent variable (control, passive, neutral, active) and planned contrasts comparing: (1) control to active responders; (2) control to passive responders; and (3) active responders to passive responders.

#### 2.1.5. Post exposure test 2: weak sucrose consumption (anhedonia)

Testing took place 10 days after the final day of fur exposure. Individual rats were trained for 2 days before the start of any testing to consume sucrose in a small plastic cage (50×35×30 cm) with a sucrose dispenser (Sippy, Ferplast, Italy) placed in one of the short walls. Training sessions lasted 10 min with access to a highly preferred 10% sucrose solution. On the test day the rats were placed in the small plastic cage for 10 min with a 1% sucrose solution available from the dispenser. The scored behavior was the volume of sucrose solution consumed by each rat. Data were analysed using one-way ANOVA with four levels of the independent variable (control, passive, neutral, active) and contrasts comparing: (1) Active responders to passive responders; (2) Active responders to control; and (3) Passive responders to control.

#### 2.1.6. Pre-exposure test and post exposure test 3: anxiety-like behavior

To assess individual differences in anxiety-like behavior before and after exposure to predatory threat, the emergence test was conducted 7 days before the first fur exposure and 15 days after the final fur exposure. This also allowed any increases in anxiety-like behavior as a result of the chronic stress exposure to be identified [39].

Testing occurred in a 120×120×60 cm wooden arena, with a black floor and white walls. A wooden hide box (40×24×17 cm), painted black with a red Perspex lid, was placed against the center of one wall. The arena was illuminated by two floodlights (with 240V, 150W globes), producing a bright open field, and a camera mounted above the arena provided video footage to a computer in another room, where behavior was scored automatically using Trackmate 5.5 (Motion Mensura, Cook’s Hill, NSW). Rats were individually tested for 5 min after being placed into the hide box and after each test the arena was thoroughly cleaned with 30% v/v ethanol. The dependent variables were: (i) time in hide box, (ii) latency to emerge, and (iii) distance travelled. Note that these behaviours are usually highly correlated, with long emergence latency associated with greater time in hide box and less distance travelled. Nonetheless, we have analysed all of them here to show the consistency of any differences in anxiety-like behaviours across all of the major behaviors examined in the emergence test. Data were analysed using two-way ANOVA with a (2) (pre-exposure, post exposure) x 4 (control, passive, neutral, active) design and planned contrasts comparing: (1) active and passive responders; (2) pre-test and post-test for active responders; and (3) pre-test and post-test for passive responders.

### 2.2. Experiment 2

#### 2.2.1. Aims

Experiment 2 aimed to assess the neural correlates of active and passive responder types and also to compare the behavioral and neural differences between rats exposed to predatory threat alone versus in a group.

#### 2.2.2. Subjects

Subjects were 32 male Albino Wistar rats (Animal Resources Centre, Perth, WA) weighing between 460 and 597 g and aged 12 weeks at the start of the experiment. Rats were housed in groups of 4 as described for Experiment 1 (see above). All rats were thoroughly handled prior to testing, and all behavioral testing took place during the dark cycle.

#### 2.2.3. Design and responder type classification

This was a 2×2 fully factorial design with 8 subjects in each of the four conditions. The independent variables were cat odor stimulus (present or absent) and exposure condition (exposed to fur either alone or in a quad).

In addition, subjects in the fur exposure conditions were classified as either active or passive using a modified version of the criteria used in Experiment 1 to account for the increased session length of 50 min. Accordingly, rats were classified as passive when they engaged in 3 or less stimulus contacts in 50 min, neutral when they engaged in 4–8 stimulus contacts and active when they engaged in 9 or more stimulus contacts. Pilot studies (unpublished findings) indicated that these slight changes to the criteria used in Experiment 1 were necessary to maintain a clear distinction between active and passive responders over the longer test session.

#### 2.2.4. Apparatus and materials

The testing arena and fur samples were the same as described for Experiment 1.

#### 2.2.5. Procedure

As in Experiment 1, all rats were uniquely marked on their backs with a Sharpie non-toxic permanent marker each day to allow identification of individual rats within a group. Prior to testing, all rats received 4 days of habituation to the test arena, but with no fur stimulus present. On the first day, half the rats were placed in one of the two arenas for 50 min alone and the other half were placed in one of the arenas in groups of 4. At the end of the habituation period rats were placed individually into small holding cages for 30 min. The following day this same procedure was repeated, but with rats that had been habituated individually on day one given group habituation and vice versa. On days three and four the procedure for days one and two were repeated. This extensive habituation procedure was used to minimise any c-Fos expression on test day due to extraneous environmental stimuli or novelty.

Testing took place over two days, with conditions counterbalanced to control for time of day effects. Subjects were placed into the arena either alone or in quads, with the cat odor stimulus either absent (no odor conditions) or flush against the center of the lower wall of the arena (cat odor conditions). Subjects were left in the arena for 50 min, after which they were removed and placed into a holding cage for 30 min. Following this they were removed from the holding cages and perfused (see below). In between test sessions the fur was heated as described earlier and the arenas were thoroughly cleaned with 30% v/v ethanol as well as a vacuum cleaner to remove any remnants of the fur, or the previous subjects.

#### 2.2.6. Immunohistochemistry

The methods used for c-Fos immunohistochemistry were as described previously (see, for example, [18], [19], [34]). Briefly, rats were deeply anesthetized then perfused transcardially. Following perfusion, brains were extracted and prepared for slicing. Tissue was sliced at 40 µm then stained for c-Fos immunoreactivity. The number of c-Fos positive cells in the regions of interest were quantified (see Table 1 for the regions and counts for areas where significant results were obtained) and images of regions of interest were prepared. A detailed description of the methods used for: tissue collection, preparation, and staining; the counting of labelled cells; and the preparation of representative images can be found in the supporting information for this paper (File S1).

**Table 1:** Mean number (± SEM) of c-Fos-positive cells in brain regions of interest.

Region	Bregma	CONTROL ALONE	CONTROL QUAD	CAT ALONE	CAT QUAD
**Sites where group vs individual** **exposure affected cat fur-induced c-Fos expression**					
AOBmc	5.70	5.29 (1.97)	9.67 (3.71)	11.33 (3.04)	19.43 (1.57)^a,b,c^
CPuM	0.70	.12 (.12)	1.12 (.40)	6 (1.49)	2.57 (.20)^a,b,c^
LPO	−0.26	1.62 (.96)	.75 (.62)	13.75 (2.37)^a,b^	8.57 (2.11)^a,b,c^
LAmg	−3.14	.5 (.76)	.37 (.37)	5.25 (1.28)	2.37 (.80)^a,b,c^
LHb	−3.14	9.87 (2.29)	8.5 (2.88)	38 (5.49)^a,b^	26.5 (4.56)^a,b,c^
DMPAG	−6.04	2.12 (.51)	1.12 (.64)	8.75 (1.06)	3.25 (2.96)^a,b,c^
**Sites where cat fur increased c-Fos expression irrespective of group vs individual exposure**					
AOV	5.20	3.62 (.65)	3 (.90)	10.29 (1.81)^a,b^	9.62 (2.87)^a,b^
MPC	3.20	2.62 (.68)	2.37 (1.08)	16.57 (4.05)^a,b^	20.75 (4.42)^a,b^
IL	3.20	5.25 (.75)	5.25 (.65)	13.71 (2.57)	12 (2.56)
AOP	3.20	2 (.91)	1.62 (.80)	5.29 (1.49)	5.62 (1.62)
LSV	0.70	11.5 (2.38)	13.88 (3.46)	37.63 (5.51)^a,b^	36.63 (5.085)^a,b^
AcbSh	0.70	.87 (.48)	1.12 (.40)	6.87 (1.41)^a,b^	7.28 (1.44)^a,b^
BSTMA	−0.26	1.37 (.82)	1.87 (.74)	9.25 (1.79)^a,b^	9.25 (2.11)^a,b^
MPA	−0.26	4 (.82)	5.87 (1.76)	11 (3.36)	11.71 (3.39)
SON	−1.30	.25 (.25)	.62 (.62)	9.12 (2.38)^a,b^	5.25 (2.37)^a,b^
PVN	−1.80	2.87 (.93)	2.27 (1.26)	18.37 (3.70)^a,b^	16.87 (2.90)^a,b^
BC	−2.12	1.12 (.79)	.87 (.40)	6.12 (2.12)^a,b^	2.87 (.51)^a,b^
CeAmg	−2.80	.12 (.12)	.75 (.37)	3.75 (1.06)^a,b^	3.87 (1.20)^a,b^
BLAmg	−2.80	.5 (.19)	.25 (.25)	4 (.98)^a,b^	3.12 (.77)^a,b^
DMH	−2.80	6.25 (1.25)	9.5 (2.37)	25.25 (3.15)^a,b^	28.5 (3.25)^a,b^
MEPV	−3.14	2 (.78)	5.25 (1.57)	25.62 (4.08)^a,b^	23.62 (2.25)^a,b^
MEPD	−3.14	1.25 (.56)	1.37 (.65)	3.62 (.92)	3.12 (.81)
VMH	−3.14–.30	0 (0)	0 (0)	19.12 (2.52)^a,b^	22.62 (4.12)^a,b^
PMD	−4.16	.25 (.25)	1.37 (.56)	65.37 (8.31)^a,b^	67 (7.55)^a,b^
VLPAG	−8.72	1.5 (.80)	1.62 (.50)	22 (2.96)^a,b^	26.87 (3.54)^a,b^
CnF	−8.72	.75 (.41)	.25 (.25)	12.12 (1.24)^a,b^	11 (1.35)^a,b^
LC	−9.68	0 (0)	0 (0)	7.14 (1.96)^a,b^	8 (1.16)^a,b^

NOTE: In all of the above regions there was a significant difference between the cat odor exposure groups, on average, and the no cat odor exposure groups, on average. a = significantly different to no cat odor alone condition; b = significantly different to no cat odor quad condition; c = significantly different to cat odor alone condition.

AcbSH = Shell of the nucleus accumbens; AOBmc = mitral cell layer of the AOB; AOP = posterior part of the anterior olfactory nucleus; AOV = ventral part of the anterior olfactory nucleus; BC = somatosensory barrel cortex; BLAmg = Basolateral amygdala; BSTMA = medial division of the anterior part of the bed nucleus of the stria terminalis; CeAmg = Central nucleus of the amygdala; CnF = Cuneiform nucleus; CPuM = Medial caudate putamen; DMH = Dorsomedial nucleus of the hypothalamus; DMPAG = Dorsomedial PAG; IL = infralimbic cortex; LAmg = Lateral Amygdala; LC = locus ceruleus; LHb = lateral habenula; LPO = Lateral preoptic nucleus; LSV = ventrolateral septum; MePD = posterodorsal part of the medial amygdala; MePV = posteroventral part of the medial amygdala; MPA = medial preoptic area; MPC = medial prefrontal cortex; PMD = dorsal part of the premammillary nucleus; PVN = paraventricular nucleus of the hypothalamus; SON = supraoptic nucleus of the hypothalamus; VLPAG = ventrolateral PAG; VMH = ventromedial nucleus of the hypothalamus.

#### 2.2.7. Data collection and analysis

Video files were given coded names unrelated to condition and were scored by an experienced blind observer using ODLog (Macropod Software). Time spent: immobile in the first 10 min of the session (rat is stationary with all four paws on the ground and no body movement); in the stimulus half of the arena; unsupported rearing (rat rears up on its hind legs without placing its forepaws on anything for support); supported rearing (rat rears up on its hind legs and places its forepaws on wall for support); and self-grooming (rat licks, scratches or face washes itself) were analysed with two-way ANOVA with the independent variables being cat odor stimulus (present or not present) and exposure (alone or group). Immobility was scored only for the first 10 min of the session as rats exposed to cat odor in a group begin to spend a substantial amount of time huddling after 10 min and it became difficult to score immobility due to obstruction by other rats.

As it was only possible to score the number of stimulus contacts when the stimulus was present, an independent samples t-test comparing rats exposed to cat odor alone to those exposed in quads was computed. Huddling, allogrooming (rat grooms another rat) and play behavior (rat pounces on, pins or chases another rat) are social behaviors and thus are only applicable to the quad exposed rats, so independent sample t-tests were computed to compare control quads to the quads exposed to cat odor. There is some debate as to whether or not adult rats engage in social play behavior, with some arguing what is classified as social play is just aggressive behavior. However, Schneider and Koch [40] argue that social play behavior occurs throughout the lifespan and simply becomes less frequent in adulthood. In the present study we have used their classification of pouncing, pinning and chasing as social play behavior.

In addition to the behaviors scored in Experiment 1, we examined the additional behaviors in Experiment 2 for several reasons. Firstly, it was of particular interest to examine immobility and grooming in Experiment 2 as the stress induced changes in these behaviors is known to be augmented by the presence of conspecifics [32], [41], [42]. Secondly, the measurement of additional behaviors was desirable so as to provide greater opportunity to identify possible links between patterns of brain activation associated with group exposure and behavior.

The number of c-Fos positive cells in regions of interest were analysed as previously described [18], [43]. Problems with homogeneity of variance were dealt with by performing a log10+1 transformation and a square root +1 transformation and selecting the transformation that resulted in the highest p value in Levene’s Test for Equality of Error Variances. ANOVAs with four levels of the independent variable (Alone no cat odor, alone cat odor, quad no cat odor and quad cat odor) were then used to compare between all groups, using the Student-Newman-Keuls procedure to control the type 1 error rate at 0.05 across all comparisons. The main effect of cat odor was compared using a planned contrast comparing the two cat odor conditions (alone and group, on average) to the two no cat odor conditions (alone and group, on average).

To compare the frequency of active and passive responders within the cat odor alone compared to cat odor group exposure conditions, a Chi-Square Test of Independence was conducted with the two variables being exposure configuration (alone or group) and responder type (active or passive). As no rats in the alone condition met the criteria for active responders, comparisons of behaviors and c-Fos immunoreactivity were made between active and passive responders within the cat odor group exposed condition using independent samples Student’s *t*-tests.

Correlations were also calculated between huddling and the number of c-Fos positive cells in the regions of interest.

## Results

### 3.1. Experiment 1

#### 3.1.1. Responder type classification

Style of responding appeared to be highly consistent: rats classified as passive overall were classified as passive on 6.1 out of 7 exposure days (87%) on average, while rats classified as active were classified as active on 5.5 out of 7 exposure days (79%) on average. Reliability analysis indicated that responder type (active or passive) was highly consistent across the 7 exposure days, Cronbach’s α = 0.928, ICC = 0.927. Neutral responders displayed a far more variable response pattern, being classified, on average, as neutral on only 2.43 out of 7 exposure days (34.7%), passive on 2.86 out of 7 (40.8%) and active on 1.71 out of 7 (24.5%).

#### 3.1.2. Fur exposure

**Figure 2:**
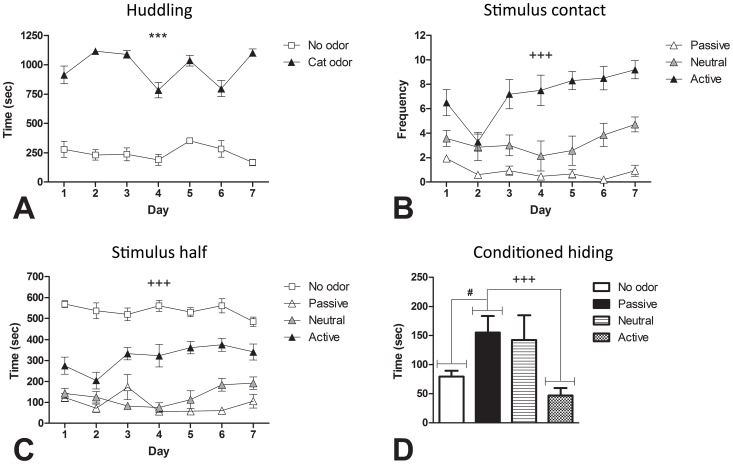
Differences between active and passive responders in the immediate and conditioned responses to cat odor. Averaged across the seven exposure days, cat odor exposed quads spent significantly more time huddling (A). Averaged across the seven exposure days, compared to passive responding rats, active responders had significantly more stimulus contacts (B) and spent significantly more time in the stimulus half of the arena (C). Active responders showed significantly less conditioned hiding when placed back in the arena 3 days after the final fur exposure with a hide box present and no fur present (D). Furthermore, relative to no odor controls, a significant conditioned hiding response was seen in passive responders, but no such response was seen in the active responders (D). *** *p*<0.001 for no odor vs cat odor; ^+++^ *p*<0.001 for active vs passive responders; ^#^ *p*<0.05 for no odor vs passive responders.

Fur exposed quads huddled significantly more than control quads on all of the 7 exposure days (see Fig 2A) (all *p*<0.001). Reliability analysis revealed the magnitude of the huddling response across the 7 days was highly consistent, Cronbach’s *α* = 0.978, *ICC* = 0.970. Averaged over the 7 exposure days, fur exposed quads (*M* = 24.8, *SE* = 1.1) deposited, on average, 11 more fecal boli than control quads (*M* = 13.9, *SE* = 2.6), *F*(1, 10) = 21.344, *p = *0.001, and spent significantly less time in the stimulus half of the arena compared to the control rats, *F*(1, 44) = 363.09, *p*<0.001.

Within the fur-exposed cohort, active responders had significantly more stimulus contacts (Fig 2B) and spent significantly more time in the stimulus half of the arena than passive responders (Fig 2C) averaged over the 7 exposure days [stimulus contacts: *F*(1, 29) = 229.67, *p*<0.001; stimulus half: *F*(1, 44) = 82.14, *p*<0.001]. Neutral responders did not differ significantly from passive responders in time spent in the stimulus half of the arena and fell between passive and active responders in number of stimulus contacts (statistics not shown).

#### 3.1.3. Post exposure test 1: Conditioned fear

When returned to the arena in the absence of cat fur and with a hide box present, passive responders spent significantly more time hiding (Fig 2D); took significantly longer to emerge from the hide box and travelled significantly less distance than the no odor control rats [hiding: *F*(1, 44) = 6.98, *p* = 0.011; latency to emerge: *F*(1,44) = 9.69, *p* = 0.003; distance travelled: *F*(1,44) = 33.13, *p*<0.001].

Conversely, there was no significant difference between active responders and the no odor control rats in the time spent hiding (*p* = 0.253), latency to emerge (*p* = 0.781) or distance travelled (*p* = 0.978). Compared to passive responders, active responders spent significantly less time hiding, took significantly less time to emerge from the hide box and travelled significantly more distance [hiding: *F*(1,44) = 12.04, *p* = 0.001; latency to emerge: *F*(1,44) = 9.10, *p* = 0.004; distance travelled: *F*(1,44) = 14.71, *p*<0.001]. Neutral responders did not differ significantly from passive responders in time spent hiding, latency to emerge, or distance travelled (statistics not shown).

#### 3.1.4. Post exposure test 2: Weak sucrose consumption (anhedonia)

**Figure 3:**
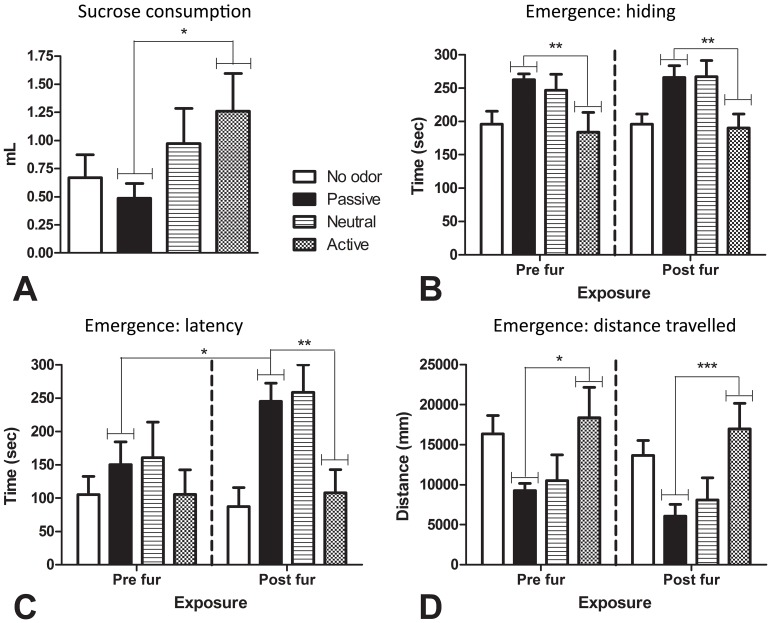
Tests of anhedonia-like and anxiety-like behavior. In the weak sucrose consumption test conducted after chronic fur exposure (A), active responders consumed significantly more of a 1% sucrose solution than passive responders. In the emergence tests, active responders spent significantly less time hiding in the pre- and post-exposure test (B). There was no significant difference in latency to emerge between active and passive responders in the pre-exposure test (C). However, there was a significant increase in latency to emerge from the pre- to post-exposure test for passive responders, but no change for active responders, with active responders having a significantly shorter latency to emerge during the post-exposure test (C). Active responders were significantly more active than passive responders during the pre- and post-exposure tests (D). * *p*<0.05; ** *p*<0.01; *** *p*<0.001.

Results of the weak sucrose consumption test for anhedonia are shown in Figure 3A. Active responders consumed significantly more 1% sucrose than passive responders, *F*(1,44) = 5.65, *p* = 0.022. There was also a trend towards active responders consuming significantly more 1% sucrose than control rats, *F*(1,44) = 3.39, *p* = 0.072.

#### 3.1.5. Pre-exposure test and post exposure test 3: Emergence test

The emergence test was conducted before and after fur exposure to test for differences in anxiety-like behavior between active and passive responders, and to examine whether exposure to a chronic stressor (7 days of fur exposure) altered this behavior. The results are shown in Figure 3B – D.

In the pre-exposure and post-exposure emergence test, active responders spent significantly less time hiding than passive responders (pre exposure: *p* = 0.007; post exposure: *p* = 0.007) and travelled greater distance (pre exposure: *p* = 0.012; post-exposure: *p = *0.001). Within-group analysis showed no significant difference between pre-exposure and post-exposure hide times or activity for active responders (hide time: *p = *0.778; *p* = 0.534) or passive responders (hide time: *p* = 0.841; activity: *p* = 0.086), although there was a trend towards passive responders being significantly less active during the post exposure test.

Conversely, there was no significant difference between active and passive responders in time taken to emerge from the hide box during the pre-exposure test (*p* = 0.379) whereas active responders took significantly less time to emerge than passive responders during the post-exposure test (*p* = 0.004). This change was due to an increased latency to emerge in passive responders: this cohort alone took significantly longer to emerge during the post-exposure test compared to the pre-exposure test (p = 0.018), while there was no change from pre- to post-exposure emergence latencies for active responders (*p* = 0.967). Neutral responders did not differ from passive responders on any of the pre- or post-exposure measures (statistics not shown).

### 3.2. Experiment 2

#### 3.2.1. Behavioral responses to cat odor that did not differ as a function of group exposure

Rats exposed to cat fur (either individually or in a quad) spent significantly less time in the half of the arena that contained the fur stimulus, showed less unsupported rearing and had significantly more escape attempts (jumps) compared to rats that were not exposed to cat odor [time in stimulus half: F(1,28) = 214.45, p<0.001; unsupported rearing: *F*(1,28) = 12.26, *p*<0.01; jumps: *F*(1, 28) = 28.27, *p*<0.001]. None of these variables interacted significantly with configuration (alone and quad) (*p*>0.2).

#### 3.2.2. Effects of cat odor exposure on huddling and social behaviors

**Figure 4:**
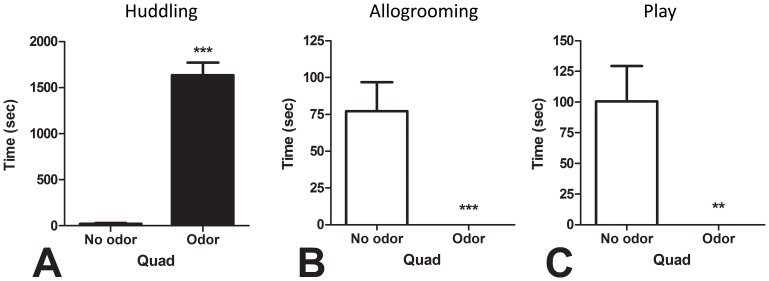
The effect of cat odor exposure on social behaviors during a 50 min exposure session. Compared to control quads, rats exposed to cat odor in quads spent more time huddling (A), and had allogrooming (B) and play behavior (C) completely abolished by the odor exposure. ** p<0.01; *** p<0.001.

Rats exposed to cat odor in a quad spent significantly more time huddling compared to the rats in quads that were not exposed to cat odor (Fig 4A), *t*(14) = 11.68, *p*<0.001. File S2 is a representative video recording of two quads from Experiment 2 demonstrating the pronounced huddling response observed in groups of four rats exposed to cat odor. Exposure to cat odor completely abolished all allogrooming (Fig 4B) and play behavior (Fig 4C) in quads of rats [cat odor quads vs control quads: allogrooming, *t*(14) = 3.92, *p*<0.001; and playing, *t*(14) = 3.48, *p*<0.01].

#### 3.2.3. Behavioral responses to cat odor modulated by social exposure

**Figure 5:**
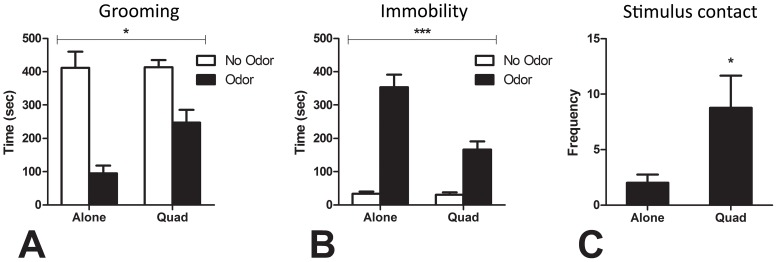
The effect of social exposure on the behavioral response to cat odor. Compared to rats exposed to cat odor when alone, rats exposed to cat odor in quads had less pronounced inhibition of grooming throughout the 50 min session (A), less pronounced induction of immobility in the first 10 min of the session (B), and made contact with the cat odor stimulus more times throughout the 50 min session. Note that the contacts (C) are only for the cat odor exposed rats as there was no stimulus present for the no odor rats to make contact with. Main effect of odor for grooming (A) and immobility (B) was significant [p<0.001], the asterisks refer to the interaction effects. * *p* for interaction effect <0.05 (A); *p* for odor alone vs. odor quad <0.05 (C); *** *p* for interaction effect <0.001 (B).

Rats exposed to cat fur spent significantly less time grooming and significantly more time immobile in the first 10 min of the session compared to rats that were not exposed to cat odor, [Grooming: *F*(1,28) = 47.96, *p*<0.001; Immobility: *F*(1,28) = 99.00, *p*<0.001]. However, the inhibition of grooming and increase in immobility in the presence of cat odor was significantly more pronounced for rats exposed alone than in a quad (Fig 5A and 5B), [Grooming: *F*(1,28) = 4.67, *p* = 0.039; Immobility: *F*(1,28) = 16.28, *p*<0.001]. Furthermore, rats exposed to cat odor in a quad had a significantly greater number of contacts with the cat odor stimulus compared to rats exposed to cat odor alone (Fig 5C), *t*(14) = 2.23, *p* = 0.043. File S3 is a representative video from Experiment 2 that illustrates the differential response of rats exposed to cat odor alone versus in a group.

#### 3.2.2. c-Fos expression

c-Fos expression in each brain region of interest for the no odor conditions (alone and quad) and the cat odor conditions (alone and quad), are presented in Table 1. In all of the regions listed in Table 1 there was a significant difference between the odor exposure groups, on average, and the no odor exposure groups, on average.

Cat fur exposure increased c-Fos expression in a number of regions irrespective of exposure condition (individual or quad). These included the ventral part of the anterior olfactory nucleus (AOV), medial prefrontal cortex (MPC), infralimbic cortex (IL), posterior part of the anterior olfactory nucleus (AOP), LSV, Shell of the nucleus accumbens (AcbSH), medial division of the anterior part of the bed nucleus of the stria terminalis (BSTMA), medial preoptic area (MPA), supraoptic nucleus of the hypothalamus (SON), paraventricular nucleus of the hypothalamus (PVN), somatosensory barrel cortex (BC), central nucleus of the amygdala (CeAmg), basolateral amygdala (BLAmg), dorsomedial nucleus of the hypothalamus (DMH), Posteroventral part of the medial amygdala (MePV), posterodorsal part of the medial amygdala (MePD), ventromedial nucleus of the hypothalamus (VMH), PMD, ventrolateral PAG (VLPAG), Cuneiform nucleus (CnF), locus ceruleus (LC), anterior part of the hypothalamus (AH) and dorsolateral PAG (DLPAG).

**Figure 6:**
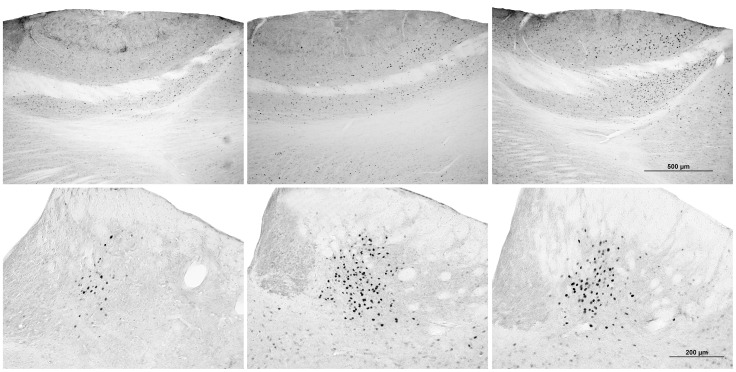
Representative images of brain slices from the mitral cell layer of the AOB (top) and lateral habenula (bottom) stained for c-Fos immunoreactivity. Mitral cell layer of the AOB: Rats not exposed cat odor (top left) had significantly less activation than rats exposed to cat odor (top middle and right). However, rats exposed to cat odor alone (top middle) had significantly less activation than rats exposed to cat odor in a group (top right). Lateral habenula: Rats not exposed to cat odor (bottom left) had significantly less activation than rats exposed to cat odor (bottom middle and right). However, rats exposed to cat odor alone (bottom middle) had significantly more activation than rats exposed to cat odor in a group (bottom right).

Cat odor exposure caused an increase in the number of c-Fos positive cells in the mitral cell layer of the AOB (AOBmc), but this effect was greater in rats exposed to cat fur in a group (see Fig 6). Conversely, the effect in the medial caudate putamen (CPuM), lateral preoptic nucleus (LPO), lateral amygdala (LAmg), dorsomedial PAG (DMPAG), and lateral habenula (LHb) was significantly lower in group-exposed rats (see Fig 6).

Finally, time rats exposed to cat odor in a quad spent in a huddle with three or four rats was significantly positively correlated with the number of c-Fos positive cells in the lateral part of the anterior olfactory nucleus (AOL) [*N* = 7, *r* = .770, *p* = 0.043], LSV [*N* = 8, *r* = 0.713, *p* = 0.047] and CPuM, *N* = 7 [*r* = 0.926, *p*<0.01].

#### 3.2.4. Differences between Active and Passive Responders

None of the rats exposed to cat odor alone met criteria for being an active responder, with one rat being classified as neutral and the rest classified as passive. Conversely, 4 rats from the quads exposed to cat odor met the criteria for being an active responder and the remaining 4 met the criteria for being a passive responder. The proportion of rats exposed to cat odor in a quad that were classified as active (50%) was significantly greater than the proportion of rats exposed to cat odor alone that were classified as active (0%), *χ^2^* = 4.31, *N* = 31, *p*<0.05.

Compared to passive responders in quads, active responders (all from quads) spent significantly less time huddling and immobile, made contact with the cat odor stimulus significantly more times, and spent significantly more time in the half of the arena containing the cat odor stimulus (see Table 2 for the means for active and passive responders for these behaviors) [huddling: *t*(6) = 3.40, *p* = 0.014; immobility: *t*(6) = 2.83, *p* = 0.03; stimulus contacts: *t*(6) = 5.13, *p*<0.01; time in stimulus half: *t*(6) = 2.56, *p* = 0.043]. There was a trend towards active responders spending more time grooming compared to passive responders, *t*(6) = 2.23, *p* = 0.067.

**Table 2:** Behavioral and neural differences between active and passive responders.

Behavioral differences, Mean (± SEM)			
Behavior		PASSIVE RESPONDERS	ACTIVE RESPONDERS
Huddling (sec)		1930 (111)	1338 (133.7)*
Immobility (sec)		216.3 (28.43)	115.9 (21.21)*
Stimulus contacts (freq)		1.75 (0.48)	15.75 (2.69)**
Stimulus half (sec)		77.86 (34.69)	193.2 (28.68)*
**Mean number (± SEM) of c-Fos-positive cells in brain regions of interest**			
**Region**	**Bregma**	**PASSIVE RESPONDERS**	**ACTIVE RESPONDERS**
Sites where active responders had significantly higher c-Fos expression			
AOBmc	5.70	16 (2)	22 (1.22)*
Sites where active responders had significantly lower c-Fos expression			
AOL	5.70	9.33 (2.33)	.75 (.48)**
LSV	0.70	48.25 (3.79)	25 (4.02)**
AcbSH	0.70	10.67 (1.45)	4.75 (1.12)*
BC	−2.12	4 (.41)	1.75 (.48)*

* *p*<0.05 versus passive responders; ** *p*<0.01 versus passive responders.

AcbSH = Shell of the nucleus accumbens; AOBmc = mitral cell layer of the AOB; AOL = lateral part of the anterior olfactory nucleus; BC = somatosensory barrel cortex; LSV = ventrolateral septum This document contains a detailed description of the procedure used in Experiment 2 to extract, prepare, slice, stain and count the tissue.

**Figure 7:**
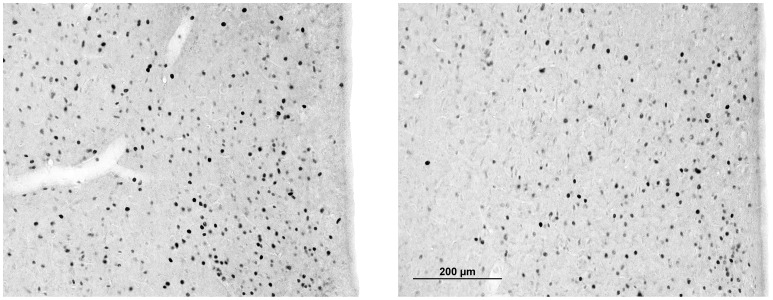
Representative images of brain slices from the lateral septum stained for c-Fos immunoreactivity. Passive responders (left) had nearly double the number of fos positive cells compared to active responders (right).

C-Fos expression in each brain region of interest for active responders and passive responders are reported in Table 2. Compared to passive responders, active responders had significantly more c-Fos positive cells in the mitral cell layer of the AOB [*t*(5) = 2.71, *p* = 0.042], and significantly fewer c-Fos positive cells in the AOL [*t*(5) = 4.22, *p*<0.01], LSV (see Fig 7) [*t*(6) = 4.21, *p*<0.01], nucleus accumbens shell [*t*(6) = 3.31, *p* = 0.021], and somatosensory barrel cortex [*t*(6) = 3.35, *p* = 0.015]. There was a strong trend towards significantly fewer c-Fos positive cells in the medial CPU of active responders compared to passive responders [*t*(5) = 2.53, *p* = 0.052].

## Discussion

This study provides some novel insights into defensive aggregation in laboratory rats using our recently developed laboratory model. As we have recently reported [13], [14], rats exposed to cat odor in a group showed a striking huddling response, and here we show that individual differences in this response and in stimulus approach are consistent over repeated exposures. The characteristic behavior of active and passive responders appears to reflect differing behavioral styles outside of situations involving acute predatory threat.

In Experiment 2, defensive aggregation was found to be associated with a social buffering effect whereby group exposure appears to promote more active styles of responding and greater performance of non-defensive behaviors such as grooming. This illustrates an important benefit of defensive aggregation that extends beyond the immediate survival advantages provided by a dilution effect. Finally, using c-Fos immunohistochemistry, we have identified brain regions associated with huddling, social buffering, and active responder styles.

### 4.1. The General Defensive Response to Cat Odor

In the present study, cat fur elicited a reliable anxiety-like response in rats exposed either alone or in a group. Fur-exposed rats avoided the half of the arena in which the fur stimulus was located, engaged in escape attempts, and spent more time immobile and less time grooming. These findings are consistent with previous demonstrations of cat odor effects in rats: including avoidance [44], [45]; immobility [45]; escape attempts [19]; and reduced grooming [18], [19], [28], [44].

Inconsistent effects of predator odor exposure on rearing have been observed, with some studies reporting increases [18], [28], others decreases [44], [46]; and others finding no effect [19] on rearing. Here we report a decrease in unsupported rearing in rats exposed to cat odor. Discrepancies may exist because lower levels of perceived threat may tend to increase rearing whereas higher levels of perceived threat inhibit rearing [47], [48]. Most studies that report an increase in rearing in response to cat odor have used a hide box paradigm [18], [28], whereas those that have reported a decrease in rearing have used either no hide box (the present study, [46]) or a hide wall that provides less concealment than a hide box [44], potentially elevating the level of perceived threat.

In addition to the changes in individual behaviors, rats exposed to cat odor in a group displayed an inhibition of social behaviors such as allogrooming and play behavior. To the best of our knowledge, this is the first report of decreased allogrooming in rats in response to predator odor; and this agrees with Louvart and colleagues [49] who reported a long term decrease in allogrooming in female rats after a single intense footshock. This fits within a general pattern of inhibition of non-defensive behaviors, such as self-grooming and play, observed in response to the cat odor in the present study, as well as previous reports of decreased play behavior in juvenile rats in response to predator odor [50], [51].

The overall distribution of cat odor-induced c-Fos immunoreactivity we observed here was consistent with previous studies [17]–[19] and highlights the role of accessory olfactory regions in the sensory processing of predator odor stimuli and the activation of characteristic downstream hypothalamic, limbic, midbrain and brainstem circuits. The LHb emerged in the current study as a site activated by cat odor, not previously reported in the literature, and very strongly activated in rats exposed to cat odor when alone (Table 1). Recent analyses suggest that the LHb may play a crucial role in suppressing locomotor activity when an aversive outcome is anticipated or when pain, stress or anxiety is experienced [52]–[54]. Thus the LHb may drive some of the avoidance behaviour, and inhibition of locomotor activity observed during predator threat in the present study, and others (e.g. [44], [45]). This may be achieved through a gating influence on midbrain dopaminergic and serotoninergic systems as well as connections from the LHb to the DMPAG, which was also strongly activated in the present study, and again more strongly in rats exposed to cat fur when alone.

The PAG has an important role in controlling the behavioral and autonomic aspects of the defensive response [54] with lesions of the dorsal PAG interfering with the behavioral and cardiovascular response to cat odor [55] as well as being associated with both escape and freezing responses to aversive stimuli [56]. The greater activation of the DMPAG in rats exposed to cat odor when alone is consistent with their different profile of defensive behavior relative to rats exposed in groups, with greater passivity, less active approach and greater inhibition of non-defensive behaviors.

### 4.2. Neural Correlates of Defensive Aggregation

This study provides an examination of the possible neural basis of defensive aggregation and shows for the first time that the propensity to huddle and the magnitude of the huddling response is highly consistent across repeated cat odor exposures. Rats that huddled more, with a passive responder style, had greater c-Fos immunoreactivity in the AOL, LSV and CPuM. The lateral septum (LS) is an area of particular interest given that it plays a demonstrable role in defensive aggregation in other species. Goodson and colleagues [6], [7] demonstrated that vasotocin V1a receptors and mesotocin in the LS potently promote flocking and flock size selection in Estrildids. It is also interesting that increasing vasopressin V1a receptor expression in the septum of Wistar rats, using viral vector mediated gene transfer, enhances their social discrimination and active social behavior [57].

The “chill of fear” [58], [59] refers to the sensation of cold that comes over people when they are fearful [60] and may possibly be related to connections between the LPO and PAG. LPO neurons are activated by cold exposure and these neurons project to the PAG where they play a crucial role in eliciting non-shivering responses to cold [61], [62]. In the present study, LPO neurons were activated by exposure to cat odor as were neurons in the PAG. It is therefore fascinating that both cold temperatures and exposure to predator odor (and, indeed, other unconditioned stressors such as bright light [13]) induce huddling, suggesting there may be some crossover in the neural substrates governing thermoregulatory huddling and defensive huddling. Notably, the LPO and PAG were more activated in rats exposed to cat odor when alone rather than in groups (Table 1).

### 4.3. Acute social Buffering

Rats exposed to cat odor in a group showed increased grooming, locomotor activity, and stimulus contacts than those exposed alone. This suggests an acute social buffering effect consistent with previous research showing the presence of conspecifics during or after a stressful event ameliorates stressor effects [32], [42], [63], [64]. For example, rats tested for contextual fear conditioning to cat odor with a non-fearful unfamiliar partner hid less, engaged in more risk assessment and had greater overall activity [65]. Kiyokawa et al. [42] reported that the presence of a conspecific reduced conditioned freezing and stress induced hypothermia to footshock, and reduced the number of c-Fos positive cells in the PVN.

Our present findings suggest a social buffering effect in groups of familiar rats that are exposed to predatory threat together. Familiarity may be a very important factor as threat induced aggregation occurs primarily between familiar conspecifics in virtually all species that huddle [1]. The social buffering effect might be directly related to a group vigilance effect [66], [67]. As a group of animals enjoys improved predator detection, individually, the animals in the group are able to devote less time to vigilance and more time to important non-threat related activities such as foraging and self-maintenance. The higher level of grooming present in socially exposed rats speaks to such an effect. This group vigilance phenomenon may operate through social buffering lessening stress, and thus freeing the animal up from vigilance-related tasks so it can continue on with other non-defence related tasks important for survival.

Several brain regions exhibited differences in c-Fos activation for rats exposed to the predator stimulus alone versus socially. Increased c-Fos expression in the AOB of socially exposed rats most likely reflects the sensory impact of the greater stimulus approach seen in socially exposed rats [18], [19]. It may also conceivably reflect exposure to alarm pheromones released by the other rats present in the social group [68].

On the other hand, social exposure to threat was associated with lesser activation in the LPO, CPuM, LHb and DMPAG. All of these regions play an important and interconnected role in threat response [52]–[54]. As noted above, the LHb is an important region that connects limbic regions such as the LPO, and parts of the basal ganglia, such as the CPuM, to the DMPAG to influence the motoric aspects of the defensive response such as immobility, cessation of non-essential activities (e.g. grooming), and avoidance of aversive stimuli [52]–[54]. Given the present finding of reduced activation in all of these regions in socially exposed rats and their more active defensive responses, future studies might explore the specific role each region has via lesion and microinfusion studies.

### 4.4. Different Responder Types

This study confirmed our prediction from informal observations that not all members of a group respond to cat fur in the same way. Specifically, we identified a clear distinction between active and passive responding. Active rats engaged in more stimulus contacts, spent more time in the stimulus half of the arena and less time immobile and huddling compared to their passive conspecifics. Importantly, the active or passive phenotype of a rat remained highly consistent across repeated exposures to cat fur. It is worth noting that on day 2 of the fur exposure in Experiment 1 there was a striking drop in the number of stimulus contacts by active responders, who then returned to the day 1 levels of contact for days 3–7. Rats exposed to cat odor individually display very similar behavior on the first and second exposure to cat odor, however, the pattern of neural activation is found to differ on second exposure, and benzodiazepines only diminish fear when given on first exposure [69], [70]. In a recent study from our group [14] there was also a decline in contacts on day 2 of group cat fur exposure, suggesting that this is a systematic effect that perhaps reflects increasing familiarity with the inescapable nature of the threat situation.

Beyond the immediate response to cat fur, active responders displayed lower anxiety and greater resilience in a number of behavioral tests. They displayed no conditioned fear to the arena in which they had been repeatedly exposed to the cat fur, whereas a pronounced conditioned fear was observed in the passive responders. Active responders also had lower levels of anxiety-like behavior in the emergence test both before and after fur exposure. Interestingly, a lasting increase in anxiety-like behavior was observed in passive responders on the emergence test following the chronic fur exposure, whereas no such increase was observed in active responders. This suggests greater resistance to the lasting anxiogenic effects of chronic stress in the active responders. Finally, active responders showed greater consumption than passive responders of a weak 1% sucrose solution post cat-odor, suggesting an absence of anhedonia in the active responders [29]. However, it is worth noting that the passive responders did not show a significant reduction in sucrose consumption relative to unstressed controls, so it remains possible that the differences in anhedonia between active and passive responders may be present irrespective of predatory stress. The behavioral correlates of active and passive responders reported here are in general agreement with other studies of active and passive coping styles in rats (for a review see [23]) and are also consistent with studies in humans which indicate passive coping strategies are associated with generalised anxiety disorder (GAD) and depression [71], [72].

When c-Fos expression was compared in active and passive responders exposed to cat odor, the active responders showed greater sensory activation in the AOB, again most likely reflecting greater approach to the cat odor source. However, they showed lesser activation in the AOL, LSV, AcbSH, BC, and CPuM. The decreased activation in the LSV of passive responders was the most pronounced neural difference between active and passive responders in the present study and is of particular interest. Previous studies involving, for example, exposure of mice to an innately aversive ultrasonic stimulus have found a similar difference between active and passive responders in the LSV [24], [73], with greater activation in the LSV in response to threat correlating with a more reactive (or passive) threat response [73].

The basal ganglia in general, and nucleus accumbens in particular, also play a role in determining whether an individual favours an active or passive coping strategy, possibly through changes in dopamine neurotransmission in this region [74]. For instance, active responding in *A. carolinensis* lizards is associated with increased dopamine levels, with increased activity of D1 receptors in the striatum as a result of the elevated dopamine thought to activate the basal ganglia to facilitate proactive responding [74]. A specific ‘defensive’ region has been identified in the nucleus accumbens with, for example, microinjections of the GABA_A_ agonist muscimol or the AMPA antagonist DNQX into caudal parts of the medial AcbSH producing fear accompanied by motivated defensive responses such as escape [75].

A key observation in the present study was that experiencing threat in a group appears to influence the distribution of coping strategies, with group exposure shifting the overall response type from primarily passive to a more equal division between passive and active responding, more closely resembling the distinct phenotypes that are observed in the wild [23]. Active responding is in some ways then a socially-mediated phenomenon as Zahavi [76], [77] suggests in his studies of the sentinel behavior of Arabian babblers. Sentinel behavior involves a babbler going to the highest part of the canopy, where they are most exposed to predators, to keep watch and alert other members of the species of a potential predator attack. Zahavi [76], [77] argues that this type of behavior has a social function, acting to elevate and reinforce social status, leading to greater access to food and reproduction. Obviously, these social benefits are not present when animals are alone, and thus the rewards of active responding are minimized and the risks maximized.

It is interesting, therefore, to consider whether these different responder types reflect a consistent inherited trait or whether there is also phenotypic plasticity. On the one hand, the active responder type only emerged during cat fur exposure in a social context, suggesting some plasticity. On the other hand, the generalization of the active responder type to other testing paradigms in which the rats were individually exposed, such as the conditioned fear test, the emergence test, and the weak sucrose consumption test, indicates trait consistency. It is therefore possible that in certain circumstances where threat is high (such as in the large open arenas in which cat fur exposure took place) the presence of conspecifics is required to allow expression of the active phenotype, possibly through social buffering and the aforementioned social motivators. Conversely, in assays that are less anxiogenic (such as the emergence test) the active phenotype is expressed in the absence of conspecifics.

### 4.5. Translational Implications

Exposure to predatory threat in laboratory animals has been seen by some authors as a possible approach for producing analogous symptoms to those of PTSD seen in humans [78], [79]. The strong conditioned fear seen in environments associated with predatory threat may be of particular interest in these models. It is therefore fascinating that active responders, in addition to their reduced anxiety and anhedonia relative to passive responders, showed an apparent lack of a conditioned fear in the testing arena where cat odor was experienced, whereas passive responders were highly anxious in this context. This suggests there is the potential for modeling susceptibility to PTSD by closely examining the active and passive responders.

The direct response to cat odor may more closely resemble symptoms of GAD and panic disorder as opposed to PTSD [80], [81]. Risk assessment behaviours (such as stimulus approach) in predator odor paradigms are thought to reflect aspects of generalized anxiety, while flight responses (such as escape attempts and fleeing followed by avoidance) suggest a response profile more similar to panic disorder [80]. The increased risk assessment and decreased freezing and stimulus avoidance in the socially exposed animals may reflect the profound benefit of social support in treating these disorders. Furthermore, the greater risk assessment and lesser freezing and stimulus avoidance in active responders than passive responders illustrates the utility of studying these distinct phenotypes to learn more about the behavioral, physiological, epigenetic and genetic factors that cause resilience or vulnerability to GAD and panic disorder.

## Conclusions

The present study suggests that defensive aggregation not only offers an immediate advantage of dilution of predatory threat, but has a buffering effect where the behavioral response to the threat is more active, and the neural response subtly altered. We confirmed our hypothesis that different, stable, coping styles exist within a cohort of rats exposed to predator threat: active responders and passive responders. Interestingly, the active phenotype emerged only when rats were exposed in a group, demonstrating the importance of social context in determining the expression of this phenotype in certain circumstances. An active style was associated with lower levels of anxiety-like and depression-like behavior and a less pronounced impact of chronic stress on behavior, as well as reduced activation in key brain regions such as the LSV while under predatory threat.

## Supporting Information

File S1 **Detailed immunohistochemistry methods.** This document contains a detailed description of the procedure used in Experiment 2 extract, prepare, slice, stain and count the tissue.
(DOCX)

File S2 **Video of the huddling response to cat fur.** This video is of the final 20 min of a 50 min session in Experiment 2 for a quad that was not exposed to cat odor (left) and a fur exposed quad (right). In the video on the right, you can see the cat fur sample placed flush against the center of the left hand wall. There is a pronounced huddling response in the fur exposed rats which is completely absent in the control rats. Note: this video has been sped up to 8x normal speed.
(MP4)

File S3 **Video of the acute social buffering effect.** This video is of the first minute of a 50 min exposure session in Experiment 2 for a rat individually exposed to cat fur (left) and four rats exposed to cat fur in a group (right). In both videos the cat fur sample can be seen placed flush against the center of the left hand wall. This video clearly illustrates the increased stimulus contacts, less pronounced fur induced immobility, and greater time spent in the stimulus half of the arena in the group exposed rats – indicative of an acute social buffering effect.
(MP4)
