# Supplementary material for: Defensive Aggregation (Huddling) in Rattus Norvegicus toward Predator Odor: Individual Differences, Social Buffering Effects and Neural Correlates
Source: PLoS One. 2013 Jul 29;8(7):e68483. doi: 10.1371/journal.pone.0068483 (PMC3726686; doi:10.1371/journal.pone.0068483)
Supplement: File S1 — Detailed immunohistochemistry methods. This document contains a detailed description of the procedure used in Experiment 2 extract, prepare, slice, stain and count the tissue. (DOCX) [file pone.0068483.s001.docx]

**Detailed Immunohistochemistry methods**

**Tissue collection, preparation and staining**

All rats were deeply anesthetized with sodium pentobarbital (Lethabarb, Virbac Pty Ltd, Milperra, NSW, Australia, 0.3 – 0.5 ml per rat), and then perfused transcardially with 200 ml of 0.1 M PBS, followed by 200 ml of 4% paraformaldehyde in PBS, pH 7.3. The brains were removed, blocked in the coronal plane, and placed in paraformaldehyde overnight at 4°C. They were then placed in 15% sucrose for 24 h, followed by 30% sucrose for 48 h. After this, the tissue blocks were placed on microtome stages, frozen to -17°C, and sectioned at 40 µm. The entire brain was sectioned coronally apart from one of the olfactory bulbs for each rat, which were sectioned in the saggital plane to allow clearer elucidation patterns of Fos in the AOB [[18](#_ENREF_18)]. Consecutive sections were placed sequentially across four vials of 0.1 M phosphate buffer (PB).

Free-floating sections were incubated for 30 min in 1% hydrogen peroxide in PB and then for 30 min in 3% normal horse serum in PB. The sections were then incubated in primary c-Fos antibody (Ab) for 72 h at 4°C (rabbit polyclonal; reacts with c-Fos p62 of mous, rat, and human; non-cross-reactive with FosB, Fra-1, or Fra-2; Santa Cruz Biotechnology, Canta Cruz, CA). The primary Ab was diluted 1:2000 in phosphate buffered horse serum (PBH) (0.1% bovie serum albumin, 0.2% Triton X-100, and 2% normal horse serum in PB). Sections wree then washed for 30 min in PB at room temperature and incubated for 1 h at room temperature in secondary Ab (biotyinylated anti-rabbit IgG made in goat; diluted 1:500 in PBH; Vector Laboratories, Burlingame, CA). They were then washed in PB for an additional 30 min and then incubated for 1.5 hr in ExtrAvidin-horseradish peroxidase (diluted 1:1000 in PBH; Sigma, St. Louis, MO). After this, they were washed three more times (30 min) in PB, after which horseradish peroxidase activity was visualized with nickel diaminobenzodine and glucose oxidase reaction as described previously [[17](#_ENREF_17)]. This reaction was terminated after 10 min by washing in PB.

The sections were then mounted on subbed slides, dehydrated in ascending concentrations of ethanol, xylene cleared, and coverslipped.

**Counting of labeled cells**

The method described above produces a black oval-shaped immunoprecipitate confined to the cell nucleus of Fos-positive cells. This was quantified microscopically at 50 sites using a brain atlas for guidance [[36](#_ENREF_36)] (see Table 1 for the regions and counts for areas where significant results were obtained). Only darkly labeled oval-shaped nuclei were counted. Three slightly different approaches to quantification were used relating to the following: (1) coronal sections for the entire brain; (2) saggital sections from the AOB; and (3) coronal sections from the main olfactory bulb (MOB).

***Coronal sections*** Coronal sections were viewed under either a 20X or 40X objective and an optical graticule was used to manually quantify the number of Fos-positive neurons in each regions. The numbers of positive nuclei that fell within a 0.5 X 0.5 mm area (20X objective) or 0.25 X 0.25 area (40X objective) in each region of interest were counted from one section per rat by an observer who was blind to group assignment.

In several cases, the designated area to be counted was substantially larger than the boundaries of the graticule. In such cases, the graticule was placed in a fixed position within the region of interest relative to known anatomical landmarks. In other cases, the designated area was smaller than the boundaries of the graticule. In such cases, only the region of interest, not the extraneous areas, were counted.

***Accessory olfactory bulb*** The AOB was quantified microscopically (40X objective) in sagittal sections. The graticule was used to quantify Fos-positive cells in 12 subregions within the anterior and posterior glomerular, mitral and granule cell layers. Each region constituted a 0.25 X 0.25 mm square and was quantified by an observer who was blind to group assignment. For statistical analysis, counts from adjacent regions were summed to provide a total of six counts per rat for the AOB. For comparison purposes, an additional count from the mitral cell layer was made from coronal sections.

***Main olfactory bulb*** For the MOB, a number (one to five) of representative coronal sections from each rat at a level of ~6.7 mm anterior to bregma were obtained. Fos expression in the entire glomerular layer of the MOB was counted microscopically for each of these sections by an observer who was blind to group assignment. For the purposes of analysis, the glomerular layer was subdivided into six separate sectors corresponding to the dorsomedial, dorsolateral, medial, lateral, ventromedial, and ventrolateral areas. A Fos count for each of these sectors was obtained for each rat by averaging counts for each sector from all the different sections counted.

For comparison purposes, two additional counts were made in the MOB in the granule cell layer using the approach described above for coronal sections.

**Preparation of images**

Digital images were made of representative pieces of tissue for illustrating the distribution of Fos-positive cells in key areas. The digitized images were produced with an Olympus DP70 12.5 megapixel camera (Olympus, Tokyo, Japan) attached to an Olympus Optical BX51 light microscope (Olympus, Tokyo, Japan). Images were acquired with a desktop computer using custom software supplied with the camera system. All images were directly imported into Adobe Photoshop CS5 (Adobe Systems, San Jose, CA, USA) and reduced in size. The only post-production enhancements were conversion of color images to black and white, the uniform adjustment of brightness and contrast for printing purposes and the whitening of non-tissue surfaces using the eraser tool.
